# Chalcogenide Perovskites and Perovskite-Based Chalcohalide as Photoabsorbers: A Study of Their Properties, and Potential Photovoltaic Applications

**DOI:** 10.3390/ma14247857

**Published:** 2021-12-18

**Authors:** Shadrack J. Adjogri, Edson L. Meyer

**Affiliations:** 1 Fort Hare Institute of Technology, University of Fort Hare, Alice 5700, South Africa; emeyer@ufh.ac.za; 2 Department of Chemistry, University of Fort Hare, Alice 5700, South Africa

**Keywords:** chalcogenide perovskites, solar cells, chalcohalide, doping, bandgap, semiconductors

## Abstract

In 2015, a class of unconventional semiconductors, Chalcogenide perovskites, remained projected as possible solar cell materials. The MAPbI_3_ hybrid lead iodide perovskite has been considered the best so far, and due to its toxicity, the search for potential alternatives was important. As a result, chalcogenide perovskites and perovskite-based chalcohalide have recently been considered options and potential thin-film light absorbers for photovoltaic applications. For the synthesis of novel hybrid perovskites, dimensionality tailoring and compositional substitution methods have been used widely. The study focuses on the optoelectronic properties of chalcogenide perovskites and perovskite-based chalcohalide as possibilities for future photovoltaic applications.

## 1. Introduction

On earth, semiconducting materials are inexpensive and plentiful. The application of these substances in solar cells for this purpose remains expedient and cheaper than photovoltaic technology based on silicon [1]. Amongst semiconducting materials, Perovskite inorganic metal halide compounds have lately obtained great attention for utilizations due to their excellent physical and chemical qualities in photovoltaics and solar cell applications [2]. Significantly, halide perovskites based on Pb have been designed to attain 29.1% power conversion efficiencies (PCEs) [3]. However, these Pb-based materials have problems with their stability, culminating in decreased long-standing performance, impeding practical applications of perovskite solar cells, and Pb toxicity problems [2]. Therefore, one of the daunting and essential research areas remains the quest for suitable solar cell materials. A new group of materials has risen in this regard; namely, chalcogenide perovskite [4].

Perovskites are a class of extremely symmetric closed-packed structure materials that have been widely researched over decades because they are versatile in their chemical and physical properties [5]. These high-class materials of perovskites have successfully increased the efficiency of perovskite solar cell systems. Recent studies have shown that the two-dimensional (2D) coated halide perovskite is integrated with the three-dimensional (3D). The extremely notable 2D perovskites are the supposed Ruddlesden–Popper (R.P.) compositions [2]. The perovskite compounds are formed from various inorganic and organic materials [6]. Besides, because of its multiple compositions and structures, they can offer a standard and enormous material-design platform, such as ABX_3_ (3D structure and three independent atomic sites), A′_2_[A_n−1_B_n_X_3n+1_] (2D Ruddlesden–Popper (R.P.) key composition), A′[A_n−1_B_n_X_3n+1_] where n is a whole number. Meanwhile, A′, A, and B designate diverse metallic positively charged ions with stable valence state, equalized through the negatively charged ion X Incorporating F^1−^, Cl^1−^, Br^1−^, I^1−^, O^2−^, S^2−^, Se^2−^, and Te^2^. In cooperation, A′ and A have to be 12 fold bonded ions to maintain a stable crystalline structure, and B is six-fold coordinated ions, with the radius of the respective ions justified by the notable Goldschmidt’s tolerance factor [7]. To estimate spatial arrangements for the perovskite crystal of ABX_3_, the factor of “Goldschmidt’s tolerance and the octahedral factor” remain widely used. The factor of tolerance is expressed as “t = (R_.A._ + R_.B._)/√2(R_.B._ + R_.X._), within which R_.A._, R_.B._, and R_.X._ are ascribed to the ionic radii of A, B, and X ions [8,9,10,11,12,13,14,15,16,17,18]. µ= RB/RX is expressed as the octahedral factor” [8,9,10].

Over the past few decades, investigations of metal chalcogenides have focused on extensive studies [19]. The expression “chalcogenide” originally comes from the term “chalcos” in Greek. For the most part, chalcogenides are compounds comprising one chalcogen element such as S, Se, or Te and either of the group IVA and VA metallic ions [20]. Oxygen, however, is not used because of its unique and enormous chemistry and must be dealt with separately [21]. Chalcogenide is a covalently bonded substance with bandgaps between 0.0 and 3.5 eV that can be amorphous or crystalline. In a similar environment, they are translucent in the infrared domain, extremely distinct from conventional inorganic and obscure glasses—for instance, silica or silicates. Chalcogenide is far less investigated than semiconducting or insulating materials due to its complex configuration, chemical constitution, and unusual interatomic coordination. The low thermal stability of the three-solid form Chalcogen constituents S, Se, and Te is evident. This led scientists to combine them with other elements to pursue chalcogenides with unique properties besides the IVA and VA elements of the group [20]. In order to develop new bonds and complexes, these three elements’ utmost valued and vital property is their resourceful disposition [22].

The transition-containing class of chalcogenide compounds and primary group metals display worthwhile physical and chemical attributes that are rationally exciting, systematically fascinating, and frequently effective for application in many technology fields. Certain examples include optical storage devices, thermoelectric devices, radiator detectors, nonlinear optics, thin-film electronics, conversion devices for solar energy, catalysis, spintronics, and even superconductivity [21]. A vast number of emerging technologies have been discovered [22].

Chalcogenides perovskites are an important group of promising, steady, and less harmful photovoltaic materials than the prevalent perovskite lead halides [23]. For Photovoltaic applications, chalcogenides perovskites in the ABX_3_ form (X = S, Se, A, B = metals with just a total oxidation state of 6) are quite environmentally safe than lead halide perovskites, have recently been suggested. Several compounds associated with chalcogenide perovskite have been so far produced. Nevertheless, CaZrS_3_, CaHfS_3_, BaZrS_3,_ and BaHfS_3_ with 3D linked corner-sharing BX_6_ octahedra are reported to occur ideally in the deformed perovskite shape. Other combinations of chalcogenide, especially from edge-sharing phases or isolated octahedra BX_6_ (the supposed ‘’need-like’’ and ‘’hexagonal’’ forms), have been experimentally synthesized. Such structures are predicted to display other localized conduction and edges of the valence band, based on the absence of related octahedra in certain crystal composition directions, and this precedes mainly heavy electrons and hole masses. Therefore, the CaZrS_3_, CaHfS_3_, BaZrS_3_, and BaHfS_3_ perovskites are predicted from carrier mobility to fit solar cell deployment [24].

Moreover, the perovskite family provides structural and compositional possibilities for exploring novel properties and applications [25]. The development of 2D Ruddlesden−Popper halogen perovskites, related to their unadulterated 2D or 3D equivalents, has drawn intense study. They have exclusive ambient stability while maintaining outstanding system performance [26]. As a result, the Ruddlesden−Popper form perovskite chalcogenide, recently demonstrated, is the ideal bandgap for a solar cell with a single-junction in a solid crystalline Ba_3_Zr_2_S_7_ [27]. Another area of interest where the perovskite family has provided rich chemical and structural possibilities is the reporting of chalcohalide perovskite that was original without lead integrated chalcogen and halogen negatively charged ions having a general formula of “A.B. (Ch, X)_3_ (A = M.A. or CH_3_NH_3_; B = Sb or Bi; Ch = chalcogen; X = halide)”. Their new properties and photovoltaic application have been developed by analyzing critical topics, for instance, structural-electronic/optical attributes and stability using various testing techniques [28]. The doping of transition metal and chalcogenide are potential ways to enhance the photovoltaic performance of the materials via bandgap engineering [23].

Perovskites are well developed to create prospects for a wide variety of photonic, optoelectronic, and energy technologies, together with that of solar cells, photoelectrochemical systems, photodetectors, and based on the combination of ultra-high absorption coefficient and projected high mobility of the carrier along with tunable bandgap, solid thermal and aqueous stability, benign and earth-abundant materials. Therefore, the study focuses on the optoelectronic properties of chalcogenide perovskites and perovskite-based chalcohalide, thus taking into account the limitations of chalcogenide perovskite and presenting solutions for them to become potential perovskites for future photovoltaic development and to estimate the photovoltaic performance of perovskite-based chalcohalide in an attempt to establish possibilities for synthesis recommendations with their perovskite-based low dimensionality of potential 3D perovskite-based chalcohalide equivalents of both MASbSI_2_ and MABiSI_2_.

### 1.1. Doping Engineering

Doping is a term that distinguishes itself from alloying theory. Light absorbers, on the other hand, are commonly alloyed and doped. Alloying is typically used to discuss metals and alloys. In contrast, doping refers to a change in the density or shape of a charge carrier caused by the addition of acceptors or donors. Both expressions have been widely used in academia [29]. In the doping phase, a contaminant known as a dopant is applied to a larger portion of the lattice (in this case, a layer of “pristine” carbon-based minute group of atoms) to alter the semiconductor’s properties [30]. Doping aims to adjust the properties of electronic, electrical, charge transport, and boundary devoid of modifying the composition of crystal and affecting the optical attributes of host materials. On the other hand, alloying is an isoelectronic positively charged ion replacement to add ionic size inequality, which could be extremely impressive for photoabsorber band engineering. Doping and alloying techniques have been critical in achieving rather high efficiencies in solar cells, using alkali components for doping and alloying with transition metals [29].

Doping involves the dopants in the silicon system contributing more electrons or holes to the Si network. Copper indium gallium selenide (CIGS) absorber occurs and is observed in halide perovskites. The change in underlying point defect concentration is changing the Fermi level. However, this doping form is tailored devoid of extrinsic dopants such as Si doping by the growth method [31]. In general, perovskites of metal halides are semiconductors with a different bandgap between valance and conduction. Synthetic doping has been rarely modified the charge transport properties of metal halide perovskites, not like metal oxides [32]. However, Phumg et al. reported halide perovskite doping by deliberate manipulation by extrinsic dopants of the material’s defect chemistry for the first time. The incorporation or segregation of dopants (Sr and Mg) will alter the pattern MAPbI_3_ material’s defect clusters. The doping of (net) n-type is either enlarged or reduced depending on the doping regime. It was incredible that, based on the concentration that varies from conventional doping, one of the dopants can implement above or below n-type doping. The edge is based on the dopant dimension of the low- and high-doping systems. The final study shows that more n-type content comes from a low doping regime, whereas high doping results in less n-type doping [31].

In the case of new electronic and optoelectronic devices, semiconductor doping is a fundamental operation. The semiconductors modification of both the optical and electrical attributes is made possible by doping, which contributes to the functionality of the primary device. The doped semiconductors’ properties describe precisely the electronic intensities initiated by the dopants. As the dopant mass increases, the detached contamination level in the host semiconductor bandgap develops into a contamination signal that may intersect through the conduction/valence band in n-/p-type semiconductors culminating in a lowering of the bandgap. The semiconductor’s optical bandgap, charge carrier mass, conductivity, and mobility regulate inorganic-organic perovskite-based optoelectronic systems’ efficiency and performance of photodetectors [33].

The potential strategies for enhancing the photovoltaic properties of lead-free chalcogenide and chalcohalide materials by bandgap engineering are transition-metal and chalcogenide doping. Simultaneously, doping in these materials can promote non-radiative charge carrier recombination, adversely affecting their photovoltaic properties. However, since both the positively charged ion and negatively charged ion doping are feasible bandgap tuning methods, it is important to test them from their effects on the lifetime of charge carriers. If, for example, a specific doping method progresses in the direction of a desirable bandgap but raises the recombination extents, it can negatively affect the photovoltaic performance of the material because it may compromise both the photocurrent and photovoltage [23]. In essence, it emphasizes that a limited number of transition metals are doped into the perovskite lattice, which then induces the processes of energy transfer and charge transfer involving the crystals and dopants. The excellent optical properties of both perovskites and transition metals are also combined by hetero-valent doping of the d block element ions into the perovskite matrix [34]. The material converting solar energy’s overall performance can be governed by several aspects: the material’s absorption coefficient (e.g., absorption spectra), chemical stability, and propensity to form defects. Elemental doping may or may not influence these factors [23].

### 1.2. Dimensionality Reduction

The formation of new substances with distinctive optoelectronic properties, including creating fertile ground for exploration, is permissible by reducing inorganic lattices’ dimensionality [35,36]. The successful general technique for adjusting semiconductor electronic structures is through dimensional reduction. There are various ways by which dimensional reduction can be accomplished in the electronic material scheme through artificial methods, for instance, superlattices, exfoliation, or thin-film development to yield 2D perovskite compounds, bonding structures that reduce the active dimensionality, interfacial electron gasses, and production of the Ruddlesden–Popper (R.P.) sequences for perovskites [37]. 2D perovskite compounds have made available novel properties, approaching innovative design conventions for system application and forming new structures [38]. Moreover, dimensional reduction produces changes associated with the electronic form of the materials, resulting in their broad range of uses and comprising, for instance, thermoelectric, where thermal power and conductivity can be decoupled, and optoelectronic components such as plain conductors, as well as a broad selection of other optical and electronic functions based on 2D compounds [37].

Due to their stability and structural flexibility, hybrid perovskites with two-dimensional (2D) layers are developing as an option to 3D equivalents, enabling fine control of these compounds’ optoelectronic properties. Theoretically, by cutting the 3D parent compounds along with a particular crystallography plane, the 2D layered perovskites are acquired, forming “perovskite slabs” that could be linked to one another by a broad range of organic positively charged ions as the restriction of size is more versatile compared to that of 3D equivalents. The responsibility of these organic cations are based on the compositional reliability of the 2D layered perovskite by electrostatic attraction in the middle of the negative charges of the halide negatively charged ions in the molecular geometry with eight faces and the positive amounts of the ammonium positively charged ions; notwithstanding the improved steady state of the 2D layered perovskite, however, further adjustments to the composition are required to evade the ultimate deterioration of the 2D compounds [39].

## 2. Chalcogenide Perovskite Photoabsorbers (ABX_3_)

Researchers were inspired to investigate chalcogenide perovskites for solar cells and other related optoelectronic characteristics by halide perovskites’ performance and promising findings of oxide perovskites in photovoltaic applications. The compositions such as “ABX_3_ (A = Ba, Sr, Ca), (B = Zr, Hf, Sn), (X = S, Se), Ba_3_Zr_2_S_7_, LaYS_3_”, and further relevant constituents are the main chalcogenide perovskites that have been investigated for optoelectronics.

### 2.1. BaZrS_3_ Chalcogenide Perovskite Photoabsorber

As a possible lead-free and steady photoabsorber, the BaZrS_3_ compound has recently withdrawn significant attention [40]. The BaZrS_3_ combination is associated with perovskite mode sulfides, which establish d^0^ diamagnetic semiconductors composed of hafnium complexes, demonstrating typically orange-brown to green colour [41]. It belongs to the perovskite family of chalcogenides “for optoelectronics functioning as photodetectors, photovoltaic, and light-emitting diodes” [42]. The following criteria were used to identify BaZrS_3_ (BZS) as one of the worthy prospects for productive photovoltaics such as: (i) it is Pb-free and can be easily synthesized; (ii) it possesses excellent carrier mobility; (iii) it is made of inexpensive, safe, Earth-rich elements; and (iii) it holds ∼1.76 eV direct bandgap, whereas the wavelength absorption is near the highest of the solar radiation spectrum [23]. Thus, amidst the chalcogenide perovskites, the highly investigated substance is BaZrS_3_. Based on the single-junction photovoltaic device, the BaZrS_3_ energy gap is less optimal such as 1.74 eV [43]. It can be tailored by positively charged ions or negatively charged ions alloying together with the fragmentary replacement of Zr atoms [44].

Regarding the preparation of BaZrS_3_, the mainstream of available data has involved bulk material (i.e., powders). For the first time, in 1957, Hahn and Mutschke formed BaZrS_3_ by heating a combination of BaS and ZrS_2_ through a closed emptied tube for a few weeks. The formation of BaZrS_3_ powders was provided by a flow of H_2_S on the mixture of BaCO_3_ and ZrO_2_, while alternatively, it is formed through BaZrO_3_ and CS_2_ sulfurization. However, by presenting BaCl_2_ and excess sulfur to BaS and ZrS_2,_ Wang et al. achieved BaZrS_3_ synthesis at temperatures as small as 450 °C, even though the maximum yield was attained at 600 °C [45].

**Figure 1:**
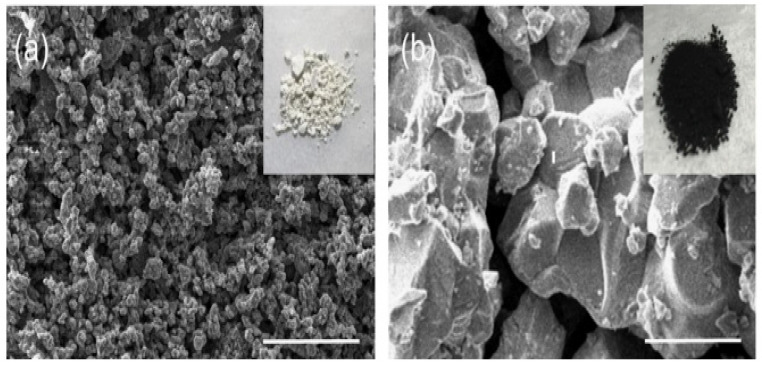
Pictures in S.E.M. of the semiconducting crystalline (**a**) Powder BaZrO_3_ applied for the formulation and (**b**) Sample of formulated BaZrS_3_ powder. “Reproduced with permission [46]. Copyright: Elsevier Ltd. (2016)”.

Perera and coworkers prepared BaZrS_3_ “by sulfurizing oxide perovskites using carbon disulfide CS_2_ at high temperatures”. The polycrystalline compound exhibited a bandgap of approximately 1.73 eV. In the visible region, the combination also showed photoluminescence (PL), confirming its direct bandgap. When compared to halide perovskites, the compounds exhibited outstanding stability under atmospheric conditions. As displayed in Figure 1, the polycrystalline SEM images show BaZrO_3_ precursor and BaZrS_3_ powder sample morphology. For the BaZrO_3_ precursor, a particle size of 100–200 nm was observed. The grains of BaZrS_3_ powder grew a considerable bigger of a little μm in size than BaZrO_3_ precursor due to sulfurization. The grains were faceted, signifying suitable crystallinity. The BaZrO_3_ exhibited a white colour, indicating no optical absorption in the visible domain, coherent with its 5eV bandgap (Figure 1a). Meanwhile, the particles of BaZrS_3_ obtained after sulfurization exhibited a black colour, signifying visible light absorption in a wide domain (Figure 1b) [46].

The synthesis of the BaZrS_3_ compound was also carried out in the thin layer with strong light absorption and elevated ambient air equilibrium [40]. Gupta et al. synthesized BaZrS_3_ by sulfurizing a thin film of BaZrO_3_, which yielded polycrystalline with an approximately 1.75 eV bandgap. In terms of stability in conditions rich in moisture, the thin film outperformed MAPbI_3_. Photodetectors were used to verify moisture-rich stability in which BaZrS_3_ photodetectors lost ≈ 40% of their first ambient response after four weeks, while related MAPbI_3_ photodetectors broke down by ≈95% in just four days [47]. However, Wei X et al. prepared thin films of BaZrS_3_ by sulfurizing oxide films lay down by a pulsed laser deposition (PLD) system at 800 °C. The films displayed remarkably intense light absorption with an absorption coefficient >10^5^ cm^−1^ at photon energy >1.97 eV. With strong carrier mobility of >13.7 cm^2^/Vs., the films are n-type and may tolerate defects with small donors from vacancies in sulfur [42].

Comparator and coworkers reported the first method of synthesizing thin films of BaZrS_3_ via sputtering at room temperature with a corresponding swift thermal procedure. Sputtering is an extensively applied industrialized method for thin film making to enormous extents. Comparatto et al. further studied temperature dependence for crystallization and the reaction progression of BaZrS_3_ film disposition. Even though temperatures needed for crystallization are relatively high, using a much longer reaction, the BaZrS_3_ thin-film effectiveness is similar to the solid-state synthesis. Considering the short fabrication time, the breadth of the XRD diffraction signals and photoluminescence response to energy and distribution showed analogous crystalline efficiency from bulk synthetic approaches [45].

**Figure 2:**
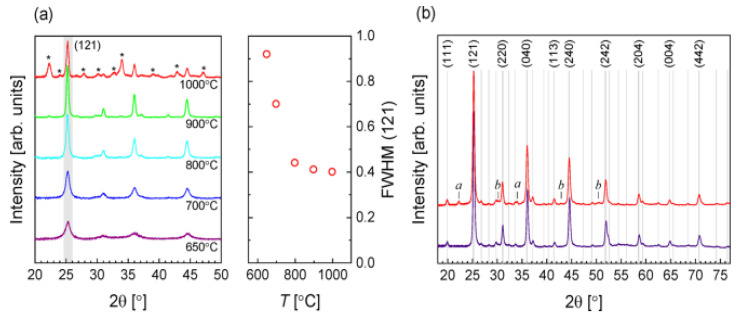
(**a**) XRD patterns (left of (**a**)) about the composition layout of classified materials being dried by starting with a temperature of 650 to 1000 °C. (right of (**a**))—the full width at half-maximum (FWHM) of the (121) peak based on the function of solidifying temperature. (**b**) With 900 °C to anneal, two samples were prepared. The lower scan is a standardized relative number of reacting particles of the materials, and the upper is a composition layout of classified materials quantified (at 180°) in the area opulent in Zr. The Clearfield et al. reference pattern for BaZrS_3_ gives rise to the Lines and indices. “Reproduced with permission [45]. Copyright: American Chemical Society (2020) with direct link as https://pubs.acs.org/doi/10.1021/acsaem.9b02428 (accessed on 30 October 2020) and further permission related to Figure 2 should be directed to A.C.S.”.

XRD was conducted to study the influence of reacting temperature on the development of crystalline phases. Figure 2a displays the designs corresponding to BaZrS_3_ for all temperatures within the 650–900 °C without evident intermediate stages. As the annealed temperature increased, the peak width (FWHM) decreased, suggesting increased crystal size and higher temperature efficiency. While the BaZrS_3_ phase’s peak width remained the same at 1000 °C, various peaks occurred in correspondence to different oxide and sulfide stages, suggesting extensive film degradation. On this basis, for further research, annealing in Figure 2b, 900 °C, was selected as an appropriate temperature, showing a detailed pattern for two samples, both annealed at the same specified temperature. The decreased pattern links to a uniform selection, with metal and sulfur material compositions similar to stoichiometry. In this example, only peaks related to BaZrS_3_ are observed. The higher pattern links with a sample classified by composition evaluated in a Zr-rich area. Several more peaks, indicated by lower case letters, are notable. In Ba–Zr–S scheme, harmonization possibly will not be made between binary or ternary sulfides. Still, they perhaps will relate to oxides: the signals labeled b (at 30.2°, 42.9°, and 50.4°) are a noble equivalent for the tetragonal ZrO_2_ phase principal diffraction peak. The labeled a could not be allocated. No additional peaks were found in the Ba-rich area [45].

**Figure 3:**
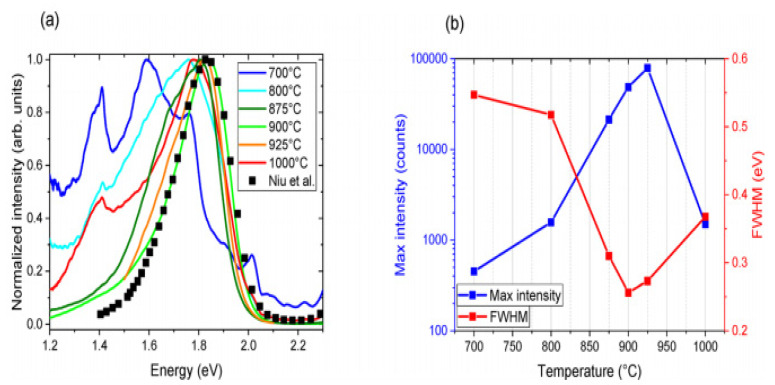
(**a**) Standardized P.L. from samples annealed at varying temperatures. Also shown with squares for comparison is the P.L. curve taken from Niu and coworkers (**b**). In the curve, the maximum intensity and FWHM are shown in (**a**). “Reproduced with permission [45]. Copyright: American Chemical Society (2020) with direct link as https://pubs.acs.org/doi/10.1021/acsaem.9b02428 (accessed on 30 October 2020) and further permission related to Figure 3 should be directed to A.C.S.”.

The most extreme PL peak for every measured temperature was chosen to understand the effect of temperature annealing. Based on the angles within the 100–135° range, which is near to the domain of theoretical analysis and in line with the relative number of reacting particles, these were mostly found; the most extreme PL curves for every temperature were obtained via plotting in Figure 3a after smoothing and normalizing along the plot reported by Niu et al. for reference. In Figure 3b, the principal signal altitude coupled with FWHM is drawn versus temperature. PL was tested at multiple spots in support of every sample and temperature annealing. Also, PL was very susceptible to the measured location because of the deposited film’s homogeneity, with a growing temperature equal to about 900 °C; PL strength at low energy decreases, while the PL signal develops stronger and smaller 1.84 eV for 900 °C annealed. Therefore, good crystallization is observed at about 900 °C, and such an elevated temperature possibly will be mismatched with the manufacture of tandem solar cells [45].

Since BaZrS_3_ is greater than the ideal bandgap for solar cells with single-junction, isovalent elements such as Ti have been successfully used for bandgap tuning by alloying. Therefore, Meng W. et al. described the preparation of BaZr_1−x_Ti_x_S_3_. A combination of BaS, ZrS_2_ and TiS_2_ with x = 0.05, 0.1, 0.2, 0.3, and 0.5 were cold compressed and melted into capsules for the formation of BaZr_1−x_Ti_x_S_3_. The capsules were loaded into quartz valves, vacuumed downward to ∼7 × 10^−7^ Torr, and the quartz valves were flame-sealed underneath a robust vacuum. The chemical reaction combinations were heated up inside a box furnace to 800 °C for 3 h and held for 15 h at this temperature. Theoretical studies have shown that a minute (∼10%) replacement of Zr by Ti (x = 0.1) could decrease the bandgap to 1.47 eV, a level inside the optimum bandgap region for solar cell technology with single-junction. It was also found that, under moderate (near the relative number of reacting particles) conditions, BaZr_1−x_Ti_x_S_3_ films should be prepared to mitigate deep-level defects development and extremely high carrier density lifetime of the minority carrier. In addition, the calculations revealed that BaZrS_3_-associated perovskite displays ambipolar self-doping attributes demonstrating the capacity to establish a homo p−n junction by adjusting growth conditions. Conversely, the theoretical studies and experimental preparation have exhibited that it would be hard to prepare BaZr_1−x_Ti_x_S_3_ perovskites because they tend to disintegrate to their subsequent ternary secondary phases. In order to develop stable BaZr_1−x_Ti_x_S_3_ alloy perovskite films, thoroughly pressured growth or non-thermal balanced growth may be required [24].

Wei X. et al. 2020 reported the synthesis of BaZr_1−x_Ti_x_S_3_ perovskite compounds by lowering the bandgap of BaZrS_3_. Ti-alloyed BaZr_1−x_Ti_x_S_3_ powders were synthesized with x mostly from 0 to 0.1. A minute Ti-alloy concentration provided a suitable bandgap whereby the BaZrS_3_ bandgap decreased from 1.78 to 1.51 eV by a (4) atom % alloy, bringing about a 32% maximum theoretical PCE for solar cells with a single junction. However, the chalcogenide perovskite phase experienced disruption triggered by the significant Ti-alloyed concentration [43]. The only way to avoid the distortion of the distorted chalcogenide perovskite from Wei et al. results is to carefully select the Ti-alloyed concentration to suppress disruption and enhance its morphology. BaZr_1−x_Ti_x_S_3_ tends to serve as a possible photoabsorber for perovskite solar cells. Synthetic methods of different kinds were used to synthesize doped and undoped doped BaZrS_3_ Chalcogenide Perovskites. They are summarized in Table 1.

### 2.2. Ba_3_Zr_2_S_7_ Chalcogenide Perovskite Photoabsorber

**Figure 4:**
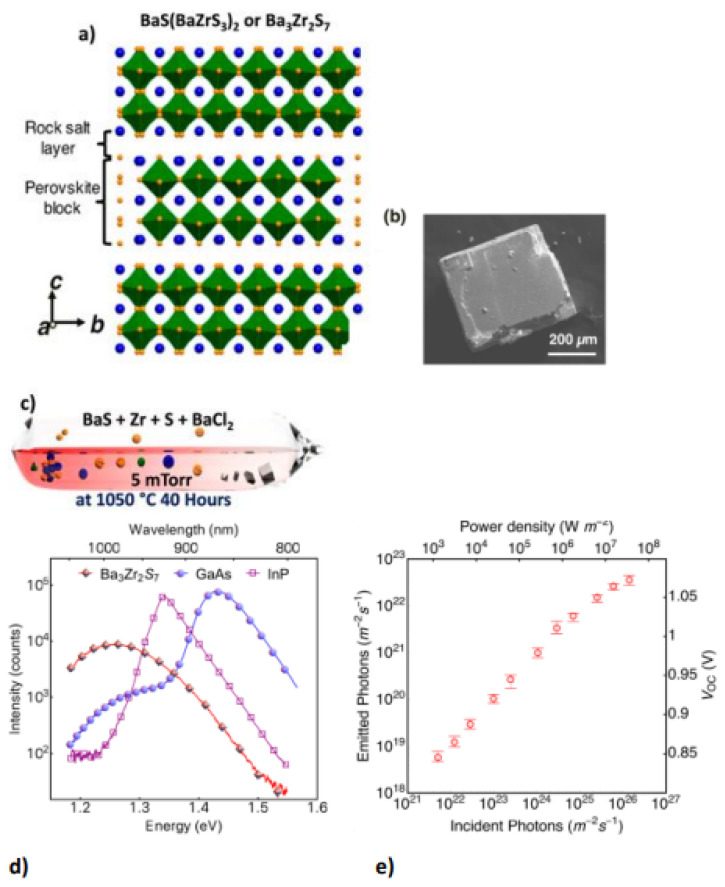
(**a**) The Ba_3_Zr_2_S_7_ schematic crystal composition. The blue and yellow balls show the atoms of the Ba and S elements. ZrS_6_ octahedrons are outlined in green. (**b**) Ba_3_Zr_2_S_7_ crystal S.E.M. representation (**c**) Schematic showing the setup of synthesis with the elevated temperature (salt flux crystal development) for Ba_3_Zr_2_S_7_ with inscription on (**c**) adapted from Ref. [4] (**d**) Under the same measuring conditions, comparison analysis of PL band of “a Ba_3_Zr_2_S_7_ crystal, an InP wafer, and a GaAs wafer are effected”. (**e**) A distinct incident power density of measurable emission and related V_.O.C_. When many sets of data have been collected, the error bar is used. “Reproduced with permission [27]. Copyright: American Chemical Society (2018)”.

Chalcogenide perovskite is a suitable photoabsorber with an ideal bandgap that would be beneficial for photovoltaic technologies. However, ferroelectricity deficiency restricts their possibility in applications [48]. Ferroelectricity or other semiconductor static polar injection could also respond to major physical influences such as shift currents. This semiconductor is the perovskite structure of Ruddlesden–Popper transition metal chalcogenides. The perovskite structure is a 2D homologous sequence of Ruddlesden–Popper phases. These coated materials can accommodate exceptional octahedral rotary motion and deformations that give rise to a noncentrosymmetric shape, a perovskite for polarized and ferroelectric attributes. 2D perovskite chalcogenide is developed by fluctuating the fixed quantity depicted as (n) of perovskite sheets with chemical formulation ABX_3_ and the sheet A.X. of rock salt, on very similar cations in the perovskite and layer of rock salt, a 2D perovskite is expressed as A_n+1_B_n_X_3n+1_ for general formula. Ba_3_Zr_2_S_7_ is the perovskite sulfide BaZrS_3_ phase of n = 2 Ruddlesden−Popper phases; two neighbouring perovskite layers intercalate one BaS layer bend-sharing ZrS_6_ molecular geometry possessing eight faces as displayed in Figure 4a. The overall surface topography of the crystals acquired were cubes and cuboids with distinct facets that surely relate to the crystal surface shown in the SEM image in Figure 4b. Theoretical and experimental studies have demonstrated that Ba_3_Zr_2_S_7_ single crystal has a 1.28 eV bandgap, favourable for fabricating single-junction solar cells. In BaCl_2_ flux sealed quartz ampoules, the distinct crystals were developed via a synthetic approach similar to BaS and Zr, which is appropriately beyond the maximum growth temperature reached in quartz ampoules and used to synthesize single-crystal Ba_3_Zr_2_S_7_. The salt flux crystal development is shown in Figure 4c [27].

For the S and Se-Chalcogen-based Photoabsorbers comparison, the different bandgaps for Chalcogenide of S and Se-based Photoabsorbers are summarized in Table 2.

The stable photoluminescence (PL) measurement upon its crystals showed an extreme and robust emission signal at 1.28 eV with 785 nm excitation at ambient temperature, which was potentially due to a change of the direct gap band to band. Figure 4d compares Ba_3_Zr_2_S_7_ radiative emission with single-crystalline InP reference and state-of-the-art wafers under the same conditions. Figure 4e displays the estimated photon flux emitted at diverse incidents of photon flux and the derived Voc beneath various illumination powers. For incident power from about 10^3^ to 10^6^ Wm^−2^, the external efficiency of luminescence is 0.1~0.15% but decreases rapidly to below 0.1% as power rises further [27].

### 2.3. SrHfSe_3_ and Sr_1−x_SbxHfSe_3_ Chalcogenide Perovskite Photoabsorbers

**Figure 5:**
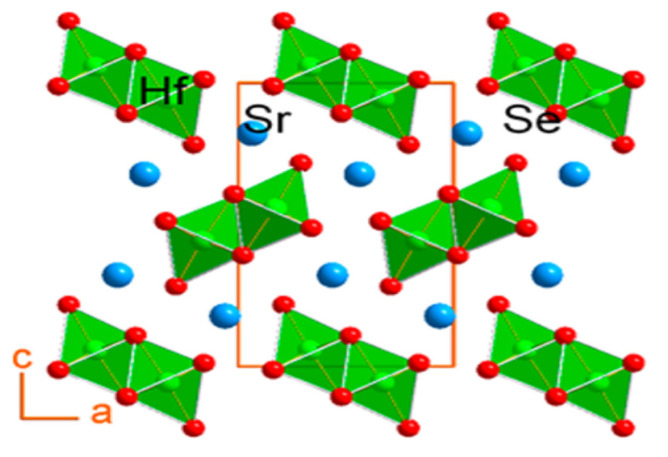
Projection to illustrate the crystal composition of SrHfSe_3_ adjacent to the b-axis, such as the octahedral geometry surrounding Hf atoms. “Reproduced with permission [49]. Copyright: American Chemical Society (2018)”.

Chalcogenide perovskite members are also single-state polycrystalline fine particles of SrZrSe_3_ and Sr_1−x_Sb_x_HfSe_3_. Using a mixture of high-temperature solid-state reactions, SrZrSe_3_ can be synthesized, and Sr_1−x_Sb_x_HfSe_3_ can be obtained from mechanical alloying. Monoz synthesized the ternary chalcogenide perovskite SrZrSe_3_ and studied its crystal composition, the optical bandgap, and thermoelectric characteristics through solid-state synthesis. With powder X-ray diffraction, the single-crystal compositional characterization showed that the prepared SrHfSe_3_ is isostructural alongside that of “SrZrSe_3_, crystallizing in the orthorhombic space group Pnma (#62) with a matrix parameter a = 8.901(2) Å; b = 3.943(1) Å; c = 14.480(3) Å; and Z = 4 for the x = 0” compositions as shown in Figure 5. The SrHfSe_3_ indicated low values of thermal conductivity data going “from 0.9 to 1.3 W m^−1^ K^−1^ and 300 to 700 K, which is more reduced by doping to 0.77 W m^−1^ K^−1^”. Electronic property measurements suggest that the compound is very insulating with a 2.9 S/cm electrical conductivity at 873 K, enhanced to 6.7 S/cm by 0.5 mol % Sb doping. Thermopower findings showed that SrHfSe_3_ is a p-type semiconductor for the 1.0 mol % Sb doping with thermopower values up to 287 μV/K at 873 K [49].

## 3. Outstanding Property Comparison of Selected Chalcogenide Perovskite Photoabsorbers

### 3.1. Optical Comparison of Selected Zr-Chalcogenide Perovskite Photoabsorbers

The optical comparison of a selected class of semiconductors with high tunability and superior optoelectronic is proposed as chalcogenide perovskites. As a result, Niu S and coworkers et al. synthesized three representatives of Zr-chalcogenide perovskites such as BaZrS_3_, α-SrZrS_3_, and β-SrZrS in two ambient temperature steadied phases, alongside iodine content to increase the rate of the chemical reaction of the solid-state method is closed ampoules. Synthetic methods of different kinds were used to synthesize Zr-chalcogenide perovskites. They are summarized in Table 3.

**Figure 6:**
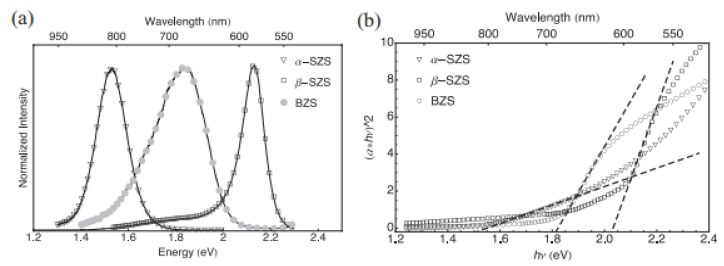
(**a**) Spectra of PL for α-SrZrS_3_ (triangles), β-SrZrS_3_ (squares), and BaZrS_3_ (circles) display respective 1.53, 2.13, and 1.81 eV bandgaps. (**b**) Using Kubelka–Munk theory, estimation of bandgap with absorption parameters recorded from diffuse measurements of reflectance and transmission on translucent powder layer. “The determined bandgap parameters are 1.52 eV for α-SrZrS_3_ (triangles), 2.05 eV for β-SrZrS_3_ (squares), and 1.83 eV for BaZrS_3_ (circles)”. “Reproduced with permission [51]. Copyright: WILEY-VCH Verlag GmbH & KGaA. Weinheim (2017)”.

PL was used to determine the optical properties of these materials at ambient temperature. As shown in Figure 6a, the PL peaks exhibited 1.53 eV bandgaps as α-SZS, 1.81 eV as BZS, and 2.13 eV as β-SZS. The theoretical values for α-SZS, β-SZS, and BZS. are 1.12, 1.73, and 1.55 eV, respectively, from computations alongside the adapted potential of Becke Johnson (mBJ). These values show a similar tendency as the investigational values but are ≈0.3–0.4 eV lower. There is a direct bandgap that exists among all the compounds. It is vital to take cognizance that the bandgap values and the absorption spectrum of α–SZS. and β–SZS. with the identical chemical substances are extremely dissimilar, suggesting the structural influence of optical properties. The bandgap was also determined using a hybrid sphere spectrometer to report absorption and dispersion using the outstanding diffuse reflectance measurements and transmittance on powder samples. The correlation involving the quantified “diffusive reflectance and transmittance estimates of absorption coefficient k and the dispersion coefficients, In (α·h-ν)2 versus h-ν plot, is given by the Kulbeka-Munk theory”. The bandgaps of the compounds were located by the absorbance value (α = kd). For α-SZS, β-SZS, and BZS, respectively, the values obtained were 1.52, 2.05, and 1.83 eV, as shown in Figure 6b. The diffuse reflectance measurements were also carried out to extrapolate the bandgaps on infinitely thick powder layers; the parameters attained matching completely through the translucent powder layers [51].

### 3.2. Thermal Stability Comparison of Selected Zr-Chalcogenide Perovskite Photoabsorbers

**Figure 7:**
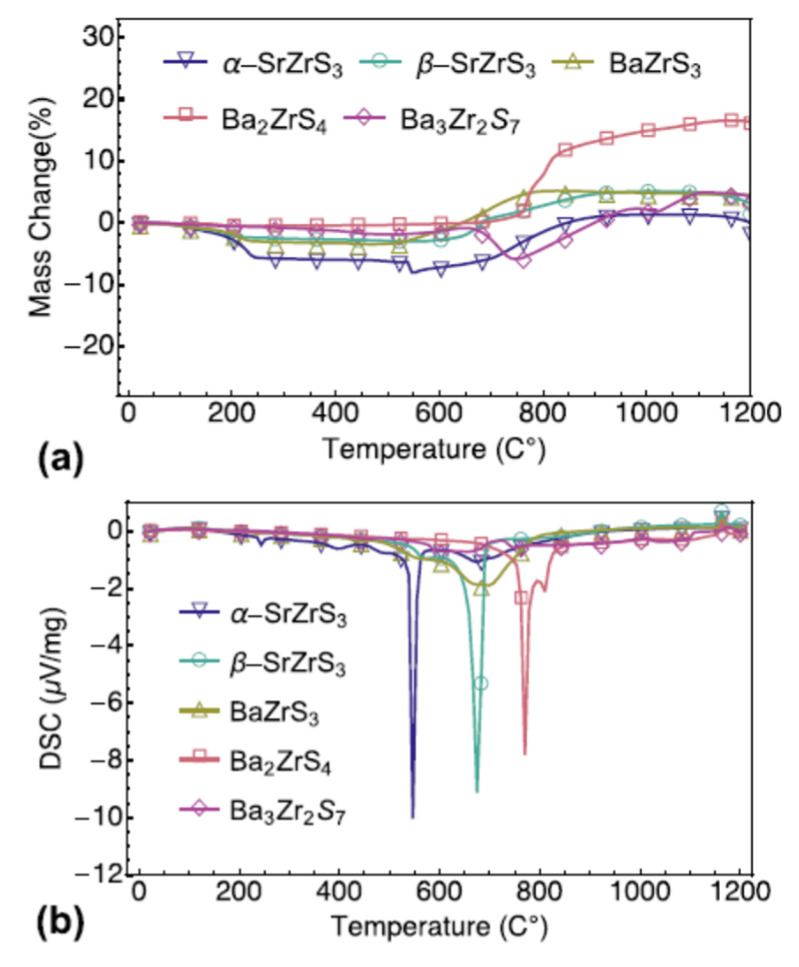
(**a**) TGA change of mass and (**b**) DSC of five samples’ temperature profiles. “Reproduced with permission [52]. Copyright: The Materials Research Society, Springer Nature Society (2018)”.

Niu S. and coworkers evaluated several chalcogenide perovskites’ thermal stability, such as BaZrS_3_ in distorted perovskite form, two SrZrS_3_ polymorphs (needle-like shape and distorted perovskite form), and two Ba_2_ZrS_4_ and Ba_3_Zr_2_S_7_ Ruddlesden–Popper phases. In sealed quartz ampoules, all samples used by Niu S. and coworkers were prepared with solid-state reaction in the high-quality polycrystalline form. Iodine was utilized to increase the rate of the chemical reaction and decrease the time of synthesis. These samples of a-SrZrS_3_ (a-SZS), b-SrZrS_3_ (b-SZS), BaZrS_3_ (BZS), Ba_2_ZrS_4_ (BZS_214_), and Ba_3_Zr_2_S_7_ (BZS_327_) were controlled at a dwell time to a cooling rate of approximately 100 °C/min using a sliding furnace setup. Moreover, “BZS., a-SZS, b-SZS, BZS_214_, and BZS_327_ samples were retained and kept at 600 °C, 850 °C, 1100 °C, 1050 °C, and 1100 °C, respectively, for 60–100 h”. For further studies, the samples collected were then grounded and pressed using a hydraulic cold press into 13 mm diameter pellets under uniaxial stress of around 600 MPa. TGA and DSC are worked on the already prepared samples in the powder phase to test thermal stability in which these compounds were heated in air to 1200 °C. Before treatment, every powder looks black or dark brown. These powders, under ambient conditions, are stable. Over year, it was observed that there had been no noticeable colour change or observable deterioration. Approximately 30 mg of powder was used for each sample and heated progressively in the air at 1200 °C. All samples were converted into white powder following the measurement. As a function of temperature, the weight change and DSC spectra are shown in Figure 7a,b; from most samples, there is a weight loss of about 200 °C, and in the DSC spectra, there are corresponding small peaks. Niu S. and coworkers attribute this to iodine evaporation, which was used to synthesize the samples as catalysis. In a-SZS compounds, this was most apparent, as those samples needed to some extent greater iodine concentrations to stabilize the preferred need-like form. All combinations remain reasonably stable in the air until well above 550 °C is heated, leading to the depletion of iodine in powder mix. The need-like stage a-SZS, with rapid weight loss and eventual progressive weight loss and related gradual weight recovery, is the first to be oxidized at 550 °C. For the two deformed orthorhombic stages, “BZS, and b-SZS, oxidation occurs at extremely near temperatures of just over 650 °C”. With oxidation onset slightly below 800 °C, the two Ruddlesden–Popper forms of BZS demonstrated the topmost stability. In their corresponding DSC spectra, “all these oxidation reactions are showed by endothermic signals”. Another exciting aspect of the TGA spectra is that a-SZS and Ba_2_ZrS_4_ showed a sharp loss of weight, accompanied by weight gain, whereas other compounds did not experience dramatic weight loss and only incremental weight gain. By replacing S with lighter atoms of O, weight loss can be comprehended. The TGA and DSC data show that a-SZS is the most susceptible to high-temperature degradation in the lower needle-like symmetry process and that all other superior methods of symmetry, including the perovskite deformed phase (BZS and b-SZS) and the Ruddlesden-popper phase (Ba_3_Zr_2_S_7_ and Ba_2_ZrS_4_), stay “reasonably stable in the air up to just over 600 °C” [52].

### 3.3. Optoelectronic Comparison of Selected Zr- and Hf-Chalcogenide Perovskite Photoabsorbers

**Figure 8:**
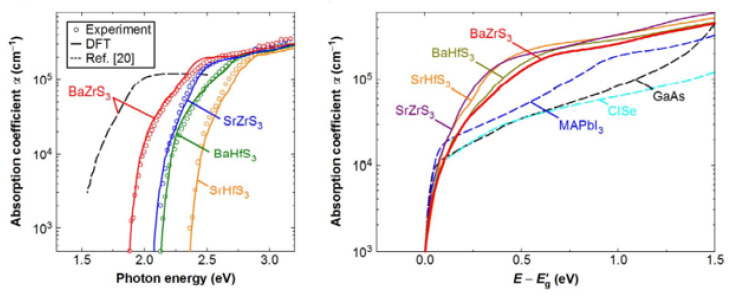
Spectra of the perovskite chalcogenides. The experimental spectra are shown by open circles show, while the solid lines reflect the DFT spectra attained through the P.H.S. process. In order to accept the stronger agreement with the experimental spectra, all the DFT spectra were blue-shifted by 60 meV. The dotted line shows the observed α spectrum recorded for a BaZrS_3_ thin layer. “Reproduced with permission [53]. Copyright: WILEY-VCH Verlag GmbH & KGaA. Weinheim (2020)”.

Studies have shown that all obtainable solar cell substances, together with hybrid perovskites, exhibit a relatively insignificant absorption coefficient (α) of approximately 10^4^ cm^−1^ in the bandgap (Eg) transition domain. The poor absorption of light on band-edged is a fundamental issue for tandem solar cells, reducing conversion efficiency. To discover the essential ability of perovskite chalcogenides as photoabsorbers of solar cells, Nishigaki et al. described the investigations on perovskite of Zr- and Hf-chalcogenides. Nishigaki et al. prepared the series of Zr and Hf-chalcogenide perovskites using a solid-form reaction method. All deformed chalcogenide perovskites such as (BaZrS_3_, SrZrS_3_, BaHfS_3_, and SrHfS_3_) were found experimentally to show unusual high absorption coefficient (α) surpassing 10^5^ cm^−1^ near bandgap (Eg,) showing the highest band-edge α amidst all identified solar cell substances, as shown in Figure 8a. The bandgap experimental values were found to be 1.94 eV for BaZrS_3_, 2.14 eV for SrZrS_3_, 2.17 eV for BaHfS_3_, and 2.41 eV for SrHfS_3_, as shown in Figure 8b. The summarized data for bandgaps of undoped and doped Zr and Hf-based distorted Chalcogenide Perovskites have been shown in Table 4. Consistent with the theoretical studies, the enormous absorption in the Eg domain rises from the strong p–d interband transition allowed by dense S 3p valence states. Low bandgap BaZrS_3_ structural analogues, Ba(Zr, Ti)S_3_ and BaZr(S, Se)_3_, were further prepared for solar cell application. In a perovskite/crystalline Si tandem of chalcogenide structural design, an earth-abundant, and safe Ba(Zr, Ti)S_3_ alloy show great potential surrounded by promising aspirants top-cell substances, attaining a maximum possible efficiency surpassing 38% [53].

**Figure 9:**
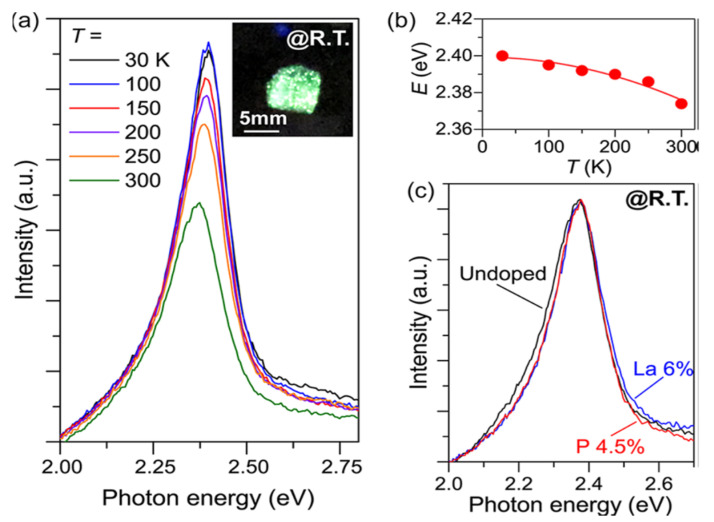
Doped and undoped SrHfS_3_ optical emission properties. (**a**) The undoped SrHfS_3_ Photoluminescence (PL) spectra were observed at 30−300 K. The inset is an image at room temperature of undoped SrHfS_3_ excited. (**b**) Dependency on the temperature of peak emission energies. The red line is a fitting result using the equation Eg = E0 − αT2/(T + β), where E0 is the Eg at 0 K, and α and β denote material constants (**c**) PL spectra of La 6%-doped (blue), P 4.5%-doped (red), and undoped (black) SrHfS_3_ at 300 K. “Reproduced with permission [55]. Copyright: American Chemical Society (2019)”.

By presenting inventive or adjusting existing electronic, optical, and magnetic properties, metal doping will broaden the role of semiconductor materials [54]. Hanzawa et al. synthesized SrHfS_3_ to attain strong carrier dopability and controllability of semiconductors to obtain optical bandgaps. Figure 9a–c shows the PL with the Temperature reliance of emission band energies for doped and undoped SrHfS_3._ The experimentally estimated synthesized SrHfS_3_ optical band gaps “were 2.3 eV (λ = 534 nm) and 2.1 eV, which relate to green and orange emissions. The SrHfS_3_ electrical conductivities were abruptly and broadly altered from 6 × 10^−7^ S·cm^−1^ at 0% to 7 × 10^−1^ S·cm^−1^ at 6% La^3+^ doping and 2 × 10^−4^ S·cm^−1^ at 4% P^3−^ doping”. At the same time, the primary carrier polarity was regulated by La doping to n-type and phosphorus doping to p-type. At 2.37 eV, both the undoped and doped SrHf_3_ displayed strong green photoluminescence (PL). SrHfS_3_ showed extreme green PL at all measured temperatures (30–3000 K) from the PL measurement, originating from band-band transition and an exciton. These electronic, optical attributes indicate that SrHfS_3_ is capable of new semiconductors that emit green light [55]. The summarized data of Hf-based Perovskite-Type Sulphide Photoabsorbers has been displayed in Table 5.

From these findings, SrHfS_3_ thin films can be developed to manufacture devices for perovskite solar cells based on the investigated specific electronic, optical properties of SrHfS_3_. Thus, SrHfS_3_ is a potential lead-free Hf-chalcogenides for perovskite solar cell application. Through the investigated unique electronic and optical attributes of SrHfS_3_, the thin films of SrHf_3_ can be formed to fabricate perovskite solar cell devices. Thus, SrHfS_3_ has the potential as a potential lead-free Hf-chalcogenide for perovskite application.

## 4. CaSnS_3_ Chalcogenide Perovskite Photoabsorber

**Figure 10:**
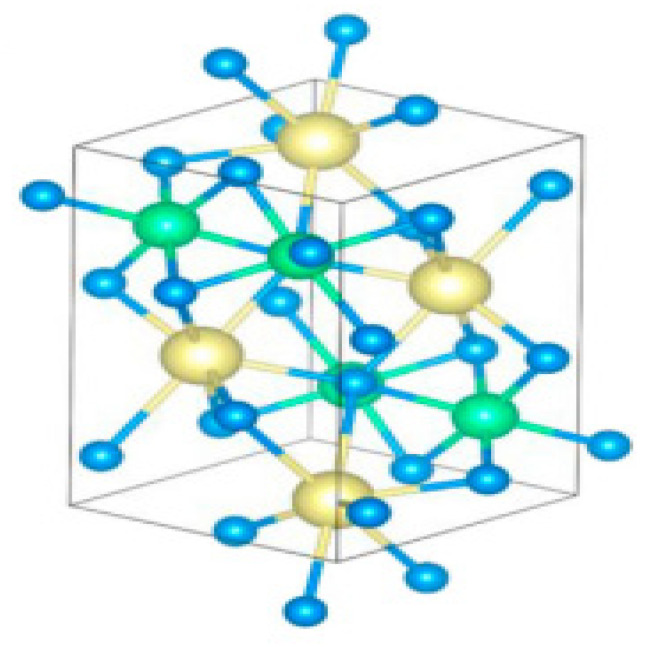
Side outlook of the geometric depiction of CaSnS_3_ matrix shaping in a deformed perovskite composition (space group Pnma). Colour code: Ca = yellow, Sn = green, S = Blue. “Reproduced with permission [40]. Copyright: Elsevier Ltd. (2020)”.

To preserve the excellent photovoltaic property and eradicate toxicity in different perovskites, Pb should be substituted by alternative nontoxic ions with lone-pair orbital such as Sn [28]. Using the ABX_3_ chalcogenide perovskite formula “(X = S, Se A, B = metals with a total valence of 6)” as stated above, the synthesized perovskite is CaSnS_3_ then (A = Ca, B = Sn and X = S) whereby Shaila et al. used an experimental route of sulfurization and deposition method to synthesize the compound. A practical route that needs an elevated sulfurization temperature and quite complicated deposition techniques were performed by preceding papers that recorded the formation of chalcogenide perovskite compounds, making it very challenging to incorporate such methods into the standard industrial fabrications. Shaili et al. carried out the synthesis through a much more exact and more straightforward process using the ultrasonic spray, considered owing to its easiness, extensive surface deposition, and inexpensive use as one of the most industrially desired methods. They reported a detailed analysis of CaSnS_3_ chalcogenide perovskite stannous-based thin films. Their main objective of reporting is to synthesize thin film of CaSnS_3_ by a facile chemical path, beginning thermal annealing with deposition of thin films of CaSnO_3_ oxide accompanied by treatment of low-temperature sulfurization. The identification by crystallography showed that the distorted perovskite composition of the CaSnS_3_ compound was successfully made, as shown in Figure 10 [40].

**Figure 11:**
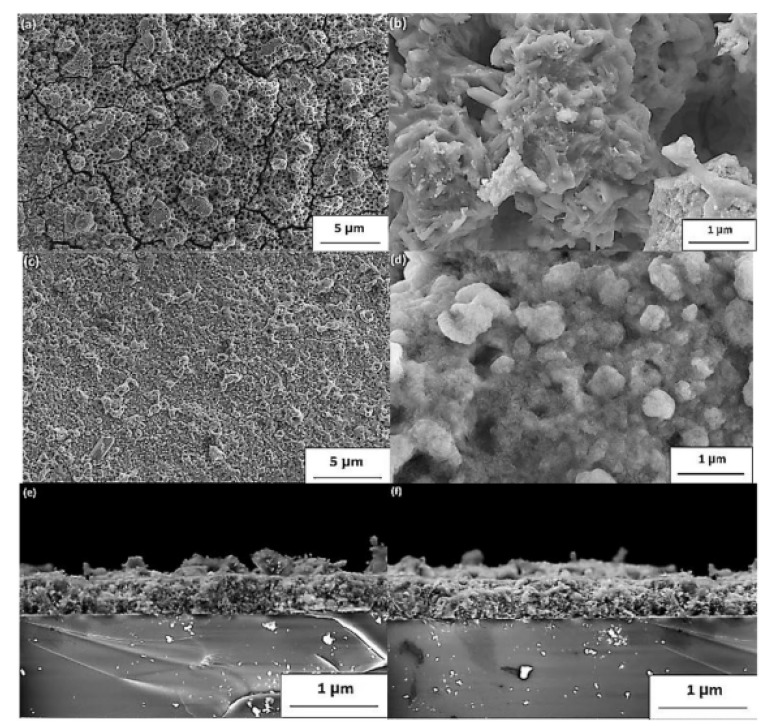
(**a**–**d**) SEM images and (**e**,**f**) cross-section view of the polycrystalline CaSnO_3_ thin films sulfurized at 400 °C (**a**,**b**,**e**) and 500 °C (**c**–**f**). “Reproduced with permission [40]. Copyright: Elsevier Ltd. (2020)”.

The FE-SEM high vacuum images of sulfurized thin layers are shown in Figure 6. From the first observation, the micrographs reveal that the film has great homogeneity with a polycrystalline disposition. Grain forming with distinct sizes can be observed at the surface level. The surface morphology was influenced by the temperature value and sulfur content, beginning with the cracks’ measure, which reduced significantly. However, the number of voids was reduced considerably for various films prepared at 400 and 500 °C, as demonstrated by the SEM images in Figure 11a–d. Besides, with only a few fractures remaining, a significant decrease in fractures was located, showing a substantial improvement in the quality of thin films. The thicknesses of the film were calculated as cross-section images at 500–520 nm, respectively, in Figure 11e–f [40].

Morphology is well established to have a basic obligation for efficiency and behaviours in halide perovskite, from the nanoscale to the macroscale. Therefore, the photovoltaics systems’ overall performance focuses significantly on material morphology, stoichiometry, and crystallinity [6]. Thus, the increasing sulfur content and temperature influenced the smoothness of the material morphology and increased the thin film’s crystallinity by reducing voids and cracks. It shows that CaSnS_3_ is a potential photoabsorber for perovskite solar application. Furthermore, Sn-based materials are commonly considered to be air-sensitive [28]. The primary challenge of rapid oxidation from divalent Sn^2+^ into more stable Sn^4+^ is usually faced by Sn-based perovskites [56,57,58,59,60,61,62,63,64]. Shaili et al. ensured that the same analysis was conducted six months after fabrication, demonstrating virtually no adjustment and excellent atmospheric stability [40]. As a result, more experiments carried out on CaSnS_3_ should be encouraged.

**Figure 12:**
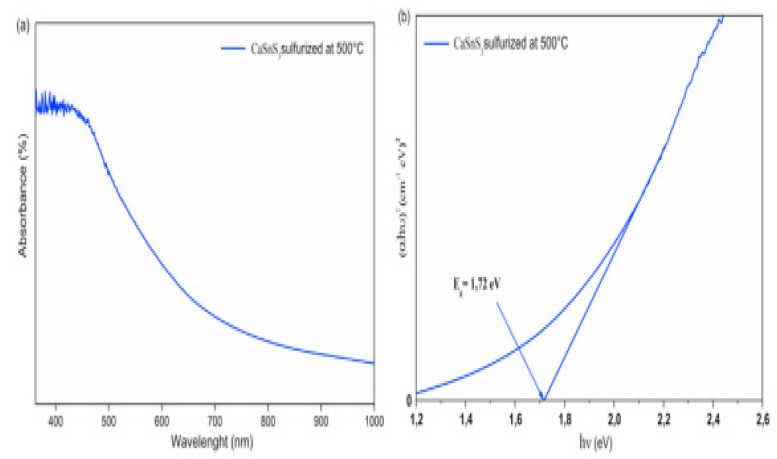
(**a**) UV-vis absorption spectrum and (**b**) bandgap of the sulfurized films at 500 °C. “Reproduced with permission [40]. Copyright: Elsevier Ltd. (2020)”.

For determining the suitability of any material as a photo absorber, the optical properties are an essential and decisive aspect, particularly the band gap value. Therefore, the CaSnS_3_ thin film using UV-vis spectroscopy in the absorption model was used to perform an extensive optical study. The investigation revealed an astounding light absorption behaviour proved by a high absorption coefficient (above 10^5^ cm^−1^) and a bandgap of 1.72 eV. It was leading to the formation of the correct phase and raising its purity. Increasing the sulfurization temperature value performed a significant function in substituting the oxygen atoms by sulfur, directing the precise stage and developing its purity. It portends a considerable influence on the material’s optical performance. Figure 12a,b illustrate the absorbance and bandgap of CaSnS_3_ thin-film sulfurized at 500 °C [40].

For determining the form of conductivity, “concentration, mobility, and resistivity of the carrier,” the measurements of Hall-effect were obtained in a room at 500 °C for the sulfurized films. A conductivity P-type with an exceptional concentration value evaluated at 1.216 × 10^17^ cm^−3^ is shown in the CaSnS_3_ thin film. The extremely high Figure of the mobility estimated at a stunning 1.314.10^2^ cm^2^ V^‒1^ s^‒1^ reveals the resulting film’s excellent crystallinity, as previously suggested by SEM images. A reasonably small value was discovered for the film resistivity, indicating a significant improvement in the film conductivity. In solar cell application, the calculated electrical properties further show the thin film’s compatibility with CaSnS_3_ as a photoabsorber [40].

## 5. LaYS_3_ Thin Film

**Figure 13:**
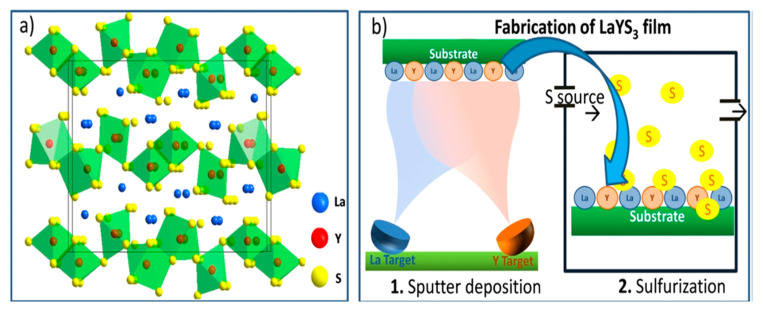
LaYS_3_ perovskite chalcogenides. (**a**) Schematic depicting LaYS_3_ crystal composition representing the perovskite structure prototype CeTmS_3_. (**b**) A schematic displays the production of LaYS_3_ thin-film supporting a two-step method involving the simultaneous sputter of the first step. “Reproduced with permission [4]. American Chemical Society (2019)”.

The LaYS_3_ is another Interesting Chalcogenide perovskite recently considered a photo absorber for heterojunction solar cell applications [40]. LaYS_3_ differs from the regular perovskites of A^2+^B^4+^S_3_^2+^. The active sites of both A- and B cations give 3+ oxidation states. In Figure 13a, the composition of LaYS_3_ is displayed. It is prepared from two-dimensional (2D) layers of [Y_3_S_9_]^9−^ stretched in the [bc] crystal plane and divided by larger ions of La^3+^. The edge-shared double-string chains of [YS_7_] trigonal prism that is monocapped (A) likewise octahedra double chains of [YS_6_] are composed of these layers (C). It establishes an ACBCA structure in which the A and B chains are shared with the C corner. Computationally, Kukar and coworkers initially discovered that LaYS_3_ preferred electronic and photonic characteristics for solar energy technologies. The first sputter deposition is La and Y (La/Y atomic ratio 1.01), and sulfurization of the resulting layer is shown in Figure 13b. Similar to a previous study of LaYS_3_ ability, analytically achieved data from X-ray and elemental study diffraction show the LaYS_3_ development in the CeTmS_3_ composition. LaYS_3_ has a film thickness of 550 nm. It is an uncommon (unless only) instance of an optoelectronically effective perovskite chalcogenide film deposition. For achieving active solar cells and optoelectronic products, it is necessary to create a good quality film. Good quality films are needed to manufacture solar cells and other optoelectronic devices [4].

**Figure 14:**
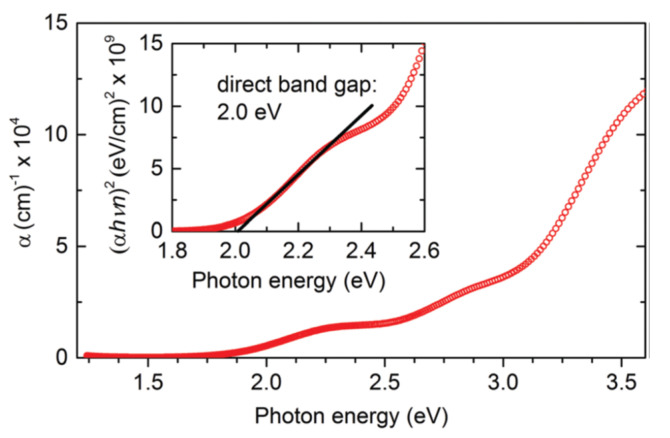
Key plots: LaYS_3_ absorption coefficient according to spectroscopic ellipsometry. Inset: Assessment of the direct bandgap value from absorption coefficient and refractive index, both obtained by spectroscopic ellipsometry. “Reproduced with permission [50]. Copyright: The Royal Society of Chemistry (2017)”.

In two synthesis steps, Kuhar and coworkers synthesized LaYS_3_ thin films. The first is the deposition on a bonded silica substructure of LaY thin-film precursor by co-sputtering metallic targets of La and Y. The second stage is quartz tube sulfurization of LaY precursor with 70 seem to flow at atmospheric pressure of 5% H_2_S in Sr. The precursors are sulfurized at 1000 °C for 10 h with a ramp rate of 101 C min^−1^ (up) and 51 C min^−1^ (down). As shown in Figure 14, this method yields a 2.0 eV bandgap [50].

**Figure 15:**
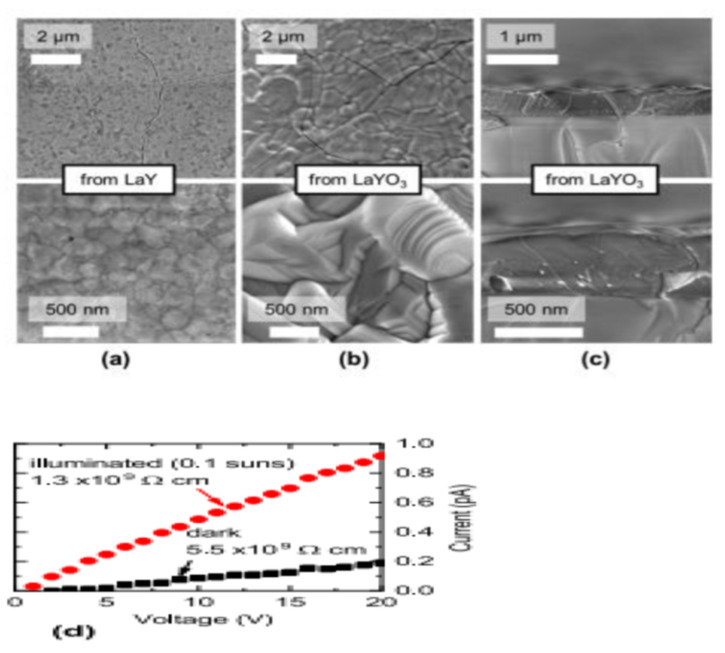
(**a**,**b**) Top-view SEM images of LaYS_3_ films derived from (**a**) metallic precursors of LaY and (**b**) oxide precursors of LaYO_3_. (**c**) Cross-sectional SEM images of LaYS_3_ films derived from precursor oxides. Top (bottom) row: images with Low (high) magnification (**d**) In-plane two-point resistivity measurement of a LaYS_3_ film at 10 mW/cm^2^ (about 0.1 suns), showing a photoconductivity effect on quartz in the dark and under white light. “Reproduced with permission [65]. Copyright: American Chemical Society (2019)”.

By sulfurizing simultaneously at 1000 °C in H_2_S, Crovetto et al. synthesized LaYS_3_ films from LaY and LaYO_3_ precursors films grown on quartz. The metallic and oxide precursors marked variants in morphology, as displayed in Figure 15. The minute particles in the diameter range of 100–200 nm smoother LaYS_3_ films were yielded by metallic precursors while the oxide precursors produced in considerably bigger particles (diameter of 1–2 μm) using advanced common roughness of the surface but often demonstrating extremely smooth surfaces, as shown in Figure 15a–c. It was observed that sulfurized LaYS_3_ films at 1000–1050 °C steadily showed band gaps about (2.0 ± 0.1) eV, but films sulfurized at or below 950 °C had greater than the bandgaps. LaYS_3_ films are photoconductive for solar power transformation technologies. When illuminated with 10 mW/cm^2^ white light (about 0.1 suns), LaYS_3_ films based on resistivity in-plane reduces by a factor of 5, as shown in Figure 15d [65].

## 6. Perovskite-Based Chalcohalide of A.B. (Ch, X)_3_ Photoabsorbers

Preceding theoretical research has revealed that the Pb lone-pair s orbital also plays a pivotal function in the friendly defect of Pb halide perovskites characteristics, apart from the three-dimensional (3D) corner-sharing lattice BX_3_ octahedra. Other nontoxic ions should substitute Pb to maintain superior photovoltaic properties and remove the harmfulness of unconventional perovskites. Pb needs to be replaced via other nontoxic ions with lone-pair energy levels, for example, Sb and Bi, to maintain superior photovoltaic properties and reduce toxicity in alternative perovskites. Suppose B is either Sb or Bi to preserve charge neutrality within the relative number of reacting particles A.B. (Ch, X)_3_. In that case, the perovskites must have mixed chalcogenides and halogen negatively charged ions, where A is alkaline earth or alkaline (or organic) positively charged ions, Ch is a chalcogenide negatively “charged ion, and X is a negatively charged ion.” B-Ch interfaces are probable to be covalent, rising to complete covalent bonding in the compound and greater atmospheric air steadiness, which is a major possible advantage of such mixed chalcogenide-halide. Previous scientific research has also revealed that the perovskite-based CH_3_NH_3_BiSeI_2_ and CH_3_NH_3_BiSI_2_ produce an adequate bandgap for photovoltaic technologies. However, chalcogenide and halogen negatively charged ions offer greater freedom for compounds and assure more excellent environmental stability. It is important to research other potential problems such as negatively charged ion ordering, phase separation, decomposition, and, if existing, address [28].

Because of benefits “such as sufficient band gaps, high absorption efficiencies, air/moisture-stability, and environmentally” benign characteristics, “antimony or bismuth chalcogenides, such as Sb_2_S_3_, Sb_2_Se_3_, Bi_2_S_3,_ and Bi_2_Se_3_ are studied to be possible photovoltaic substances”. Although the PCEs of solar cells based on “antimony or bismuth chalcogenides are substantially lower than organic−inorganic lead halide, they display comparatively healthy air/moisture-stability, which is crucial PSCs” [66]. The integration of chalcogenide negatively charged ions based on the halide bismuth perovskites will minimize their bandgaps without impinging on its optoelectronic properties. Another possible benefit of adding chalcogen negatively charged ions is that the Bi-chalcogen bonds are more covalent than the Bi-halogen bond, resulting in a greater importance of the whole compound covalence significance, resulting in more excellent stability. Computational predictions indicated a steady form of the mixed chalcogen-halogen perovskite, which merited photovoltaic advancement [67]. The Summarized data for device performance of Perovskite-based Chalcohalide photoabsorbers has been displayed in Table 6.

### 6.1. Perovskite-Based Chalcohalide of MASbSI_2_ Photoabsorber

**Figure 16:**
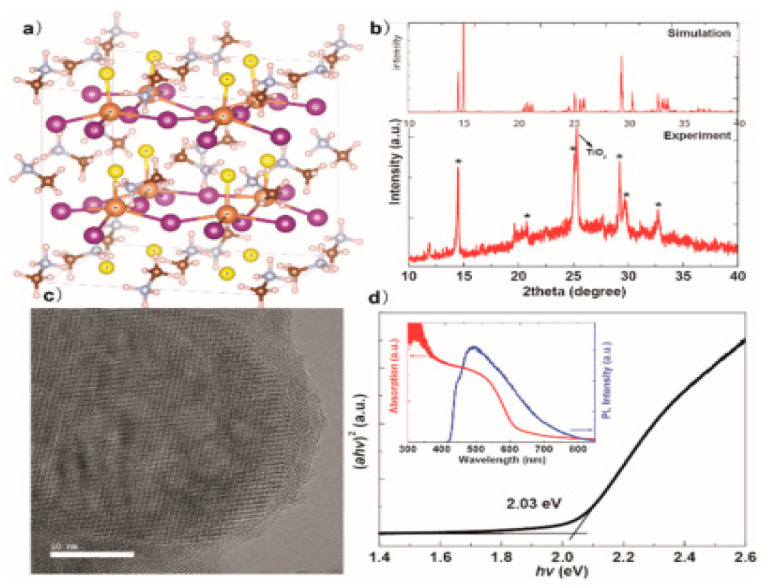
(**a**) The ball presents MASbI_2_S perovskite-based chalcohalide simulated DFT and the orange ball stick prototype composition “for Sb, purple for I, yellow for S, gray for C, brown for N, and white for H. (**b**) Simulated and empirical comparison of XRD pattern (**c**) TEM/TiO_2_/ MASbSI_2_ Glass High-resolution (**d**) Tauc’s glass/TiO_2_/MASbI_2_S plot. Inset: the related UV−vis absorption spectrum and PL spectrum”. “Reproduced with permission [66]. Copyright: American Chemical Society (2018)”.

In a perovskite structure, the use of bivalent chalcogenides and univalent halides as negatively charged ions enables the incorporation of 3^+^ and 4^+^ positively charged ions in the position of 2^+^ metal positively charged ions. Nie and coworkers first reported developing solar cells using the composition of MASbSI_2_ perovskite-based chalcohalide as photoabsorbers. Under mild conditions, the MASbSI_2_ perovskite-based chalcohalide was prepared by annealing by a progressive reaction concerning antimony trisulphide (Sb_2_S_3_), which is placed on a mesoporous TiO_2_ electrode by the chemical bath deposition (CBD) process, also precursors such as SbI_3_ and MAI and then annealed at 150 °C in argon surroundings. The enhanced crystal composition of the MASbSI_2_ perovskite-based chalcohalide is shown in Figure 16a, with sulfur atoms distributed in the octahedral unit along the X-axis. “Sb−S bonds were divided into lengthier (∼3.6 Å), and smaller (∼2.27 Å) bonds, and ∼3.16 Å was the length of the Sb−I bond” [66].

As shown in Figure 16b, the empirical XRD designs corresponded with the computer-generated one, suggesting the viability of phase synthesis of perovskite-like. As shown in Figure 16c, the high-resolution TEM observed the formation of well crystalline MASbSI_2_ perovskite-based chalcohalide. The MASbSI_2_ perovskite-based chalcohalide bandgap, achieved “from the Tauc plot using the UV-visible absorption spectrum was 2.03 eV, with an absorption edge of about 600 nm”, as shown in Figure 16d. The MASbSI_2_ perovskite-based chalcohalide photoluminescence spectrum was seen inside. The PL signal did not align well with the absorption edge, which could be based on the oscillating alloy capability of indirect bandgap alloys. Those observed in previous literature are close to the phenomenon. The construction of solar cells through MASbSI_2_ perovskite-based chalcohalide exhibited a photocurrent of 8.12 mA/cm^2^, a value of 0.65 V as a voltage source and fill factor (F.F.) of 58.5%, thereby giving rise to a PCE of 3.08% [66].

### 6.2. Perovskite-Based Chalcohalide of MABiSI_2_ Photoabsorber

Bismuth is highly preferred among other lead-free substitutes, for instance, copper, germanium antimony, tin, and alkaline-earth metals. Due “to its low trap state densities, long carrier lifetimes,” besides the functional capacity to withstand defects as expected based on theoretical and empirical studies, and more significantly, Bi is less harmful than Pb. While bismuth perovskite showed exhilarating succession, main bismuth perovskite difficulties have also been revealed. Bismuth perovskites will crystallize, supporting intrinsic defect sites, inside a 2D hexagonal layered form. Another problem is that, for perovskite solar cells, bismuth appears to possess a sizable and indirect bandgap around 2.0 eV, whereas the bandgap is not extremely appropriate. Although bismuth chalcogenides have been described as lesser bandgaps than bismuth halide perovskites such as Bi_2_S_3_ and Bi_2_Se_3_, these compounds are stable and safe. Zhang et al. concluded that the introduction of chalcogenides would reduce their bandgaps of halide perovskites. Hence, Zhang et al. was the first to synthesis new lead-free Methylammonium bismuth sulfur diiodide (MABiSI_2_) as photoabsorbers and established its physical and optical properties by numerous analysis techniques [67].

**Figure 17:**
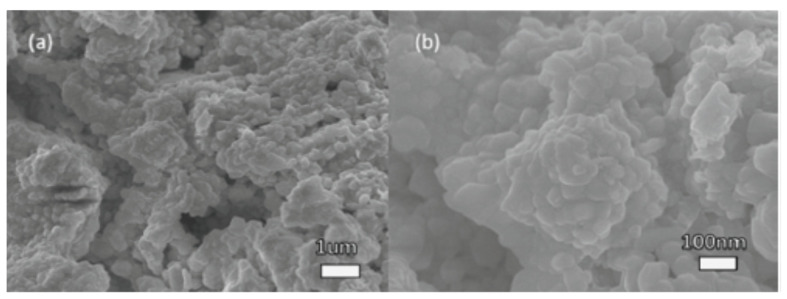
SEM images of the MABiSI_2_ perovskite-based chalcohalide sample. (**a**) Picture for the general outlook of the sample powder (**b**) The nanoparticles nearby appearance. “Reproduced with permission [67]. Copyright: The Chemical Society of Japan (2019)”.

Zhang et al. prepared a new and high crystallinity MABiSI_2_ perovskite-based chalcohalide using a two-step solid-state reaction phase. The SEM images of MABiSI_2_ perovskite-based chalcohalide in which MABiSI_2_ perovskite-based chalcohalide was grown in approximately 50 nm minute 3D nanoparticles are presented in Figure 17a,b [67].

**Figure 18:**
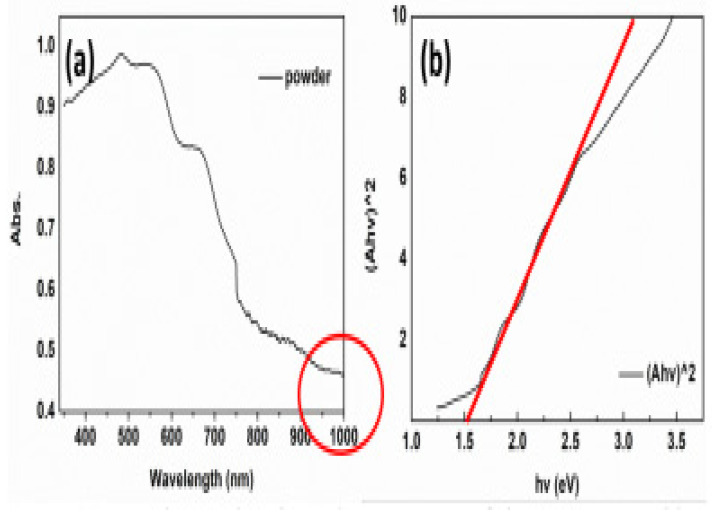
(**a**) MABiSI_2_ perovskite-based chalcohalide UV-vis powder absorption spectrum with an absorption domain attaining up to1000 nm. (**b**) The Tauc plot is originated from the MABiSI_2_ perovskite-based chalcohalide absorption spectrum of UV-Vis. “Reproduced with permission [67]. Copyright: The Chemical Society of Japan (2019)”.

To test the potential of the MABiSI_2_ for light absorption, Zhang recorded the UV-Vis spectrum. The MABiSI_2_ sample powder UV-Vis absorption spectrum is displayed in Figure 18a. From the Figure, MABiSI_2_ perovskite-based chalcohalide has started to exhibit 350 nm optical absorption and has a high absorption band in the 350–700 nm range. The edge of absorption was increased to 750 nm whereas yet retaining half of the full intensity. In the domain of visible light, which is proportionate to perovskite Pb-based, it can be deduced that the MABiSI_2_ perovskite-based chalcohalide shows an extremely favourable light-absorbing ability, offering intense absorption and broad absorption limit. “In the infrared region of the solar spectrum,” the MABiSI_2_ perovskite-based chalcohalide displayed a proper absorption efficiency, extending its absorption region to more than 1000 nm. The unique absorption region will be useful for single-junction solar cell applications and multi-junction device applications. The Tauc plot was constructed with the UV-Vis absorption spectrum aid, as shown in Figure 18b. By following the relationship below, the MABiSI_2_ perovskite-based chalcohalide bandgap was estimated. The optical bandgap of MABiSI_2_ perovskite-based chalcohalide was obtained to be 1.52 eV by extrapolation. For photovoltaic devices, this is a very promising bandgap attribute. The new perovskite material can be applied to the solar device, judging by the optical properties above. The MABiSI_2_ perovskite-based chalcohalide material exhibited a high defect tolerance ability. Its unique properties showed that it could be a worthwhile photoabsorber with superior system manufacturing methods. Solar cells fabricated with the MABiSI_2_ perovskite-based chalcohalide were examined to have “a PCE of 0.13%, a photovoltage of 0.22 V, resultant current of 1.96 mA.cm^−2^, and fill factor of 0.30” [67].

## 7. Challenges and Future Trend of Chalcogenide Perovskite and Perovskite-Based Chalcohalide Materials

Intensive research has been performed on the proposed solar cell materials since introducing chalcogenide perovskites as potential solar cell materials. There are study gaps, however, which need to be discussed. This section is dedicated to defining the problems and presenting solutions for their optoelectronic application to overcome them. The Perovskites could be called semiconductors of a soft ionic solid with attributes and several pervasive behaviours, such as ion migration, ferroelectricity, hysteresis, vibrational matrix characteristics controlling the transport of charge carriers. Related to whatever has been discovered in inorganic and organic semiconductors, defects as wells as perovskite-based solar cells’ overall performance and effectiveness play an important role [68].

Under an applied electric field, ferroelectric materials do something unique: they show spontaneous polarization, indicating that positive and negative charges are easily separated within the crystal. This property makes the material suitable for energy conversion and storage [69]. For Photovoltaic devices, chalcogenide perovskites with ideal bandgaps and favourable light absorption are promising, while the ferroelectricity paucity constrains the utilization ability. Based on theoretical studies, it was discovered that the core pathway based on the paraelectric character of Ba_3_Zr_2_S_7_ was identified in experiments and showed a common control aimed at ferroelectricity growth in A_3_B_2_X_7_ Ruddlesden-Popper (R.P.). Group theoretical analysis indicates that the primary feature that dominates ferroelectricity is the tolerance factor. Due to in-phase rotation suppression, both Ba_3_Zr_2_S_7_ and Ba_3_Hf_2_S_7_ have high tolerance factors. As a result, they are paelectric and vital for inappropriate ferroelectricity.

The Ca_3_Hf_2_S_7_, Ca_3_Zr_2_Se_7,_ and Ca_3_Hf_2_S_7_, in comparison, demonstrate in-phase rotation with limited tolerance factor and can be steady with non-trivial polarization in the ferroelectricity ground state. These outcomes afford relevant ground rules for the engineering of R.P. chalcogenides ferroelectricity and suggest possible semiconductors of ferroelectricity for photovoltaic applications [48].

Although studies have shown that chalcogenide perovskites and associated compounds are an exceptional and promising group of optoelectronic substances, several fundamental material properties include the optical absorption defect property carrier, concentration, and mobility coefficient, most of which remain lacking. This major setback is because most studies have concentrated on powder or single-crystal bulk samples to a great extent involving the absence of a thin layer. The thin layer scarcity of samples limits our basic knowledge and becomes an obstacle to applying photovoltaic systems. Although Wei X et al. has described the development of BaZrS_3_ thin films to estimate its carrier densities, mobility, and absorption coefficient measurements, thereby these findings could theoretically release the group of perovskite chalcogenides for optoelectronics, for instance, photovoltaics, photodetectors, and light-emitting diodes, ensuring that BaZrS_3_ is a promising candidate [42]. However, this is an excellent step in the correct direction, but based on these crucial parameters for the actualization of efficient optoelectronic application, many more studies need to be performed on many other chalcogenide perovskites.

Device manufacturing essentially relies on the characteristics of the thin layer. High-quality layers have to be produced and their optoelectronic properties studied. The processed film growth method of a simple solution is an important justification for the rapid development of optoelectronics based on halide perovskite. Currently, the prospect of solution-processed film processing perovskite chalcogenide is cloudy due to the synthetic method. Significant advances are possible if solution-processed chalcogenide perovskite synthesis can be achieved. Supplementary film manufacturing techniques such as sputtering should be investigated in the lack of this solution-processed form. The sputtering process developed the LaYS_3_. However, in such a sputtering method, maintaining exact stoichiometry may become a problem. Therefore, rigorous characterization of such film is substantially required [4].

Meanwhile, the lead-free chalcohalide materials range from visible to UV bandgaps, and many exhibits ferroelectric activity at lower temperatures, whether photoconductive or photovoltaic effects containing Sb and Bi elements [70]. The MASbSI_2_ tolerance factor calculated is 0.99 [66], and that of MABiSI_2_ is 0.853, in which both were near to the (1.0) basic cubic perovskite configuration. The MASbSI_2_ and MABiSI_2_ capacity to produce a simple 3D perovskite composition has been demonstrated. The MABiI_2_S material exhibited high defect tolerance ability and exhibited a PCE of 0.13% [67]. A PCE as high as 3.08% exhibited the best-performing MABiSI_2_—based solar cells. However, the latest lead-free perovskite-based chalcohalide materials from MASbSI_2_ and MABiSI_2_ and their uses in photovoltaic technologies, for instance, solar cells, have been devoted to considerable efforts. Recent focus has not been assigned to low dimensional perovskites, comprising two-dimensional (2D), one-dimensional (1D), and zero-dimensional (0D) lead-free chalcohalide materials. The crystalline lead-free chalcohalide materials with lowered dimensionality show unique optoelectronic properties, just like their metal halide perovskites counterparts. Furthermore, as studied in compositional replacement of ABX_3_ halide perovskites, there is no uncertainty that the replacement of positively charged ions (A) and metal (B) in the composition of chalcohalide lead-free materials will redefine its bandgap and widen the emission spectrum, alongside the anion mixing technique, to improve bandgap tuning [34]. Therefore, it is of vital importance that dimensionality tailoring and compositional replacement techniques should be integrated into the lead-free chalcohalide perovskites to establish new synthesis and characterization of novel lead-free chalcohalide materials with reduced dimensionality and compositional replacement for distinctive optoelectronic properties.

## 8. Conclusions and Prospects

The research herein focused on the properties of lead-free chalcogenide and chalcohalide perovskites. Thus, we considered the drawbacks of the lead-free chalcogenide and offering alternatives for them to become potential perovskites for future photovoltaic development and evaluate the photovoltaic performance of lead-free chalcohalide to introduce opportunities for 3D lead-free chalcohalide equivalents and their low-dimensional perovskites to be synthesized.

Chalcogenide perovskites with ideal bandgap and favourable light absorption promise photovoltaic systems, though the lack of ferroelectricity limits their application capability. Based on theoretical studies, it was found that the core pathway of the paraelectric Ba_3_Zr_2_S_7_ character was performed in investigations and that a common law on behalf of the presence of ferroelectricity in Ruddlesden-Popper (R.P.) A_3_B_2_X_7_ was demonstrated. Group theoretical analysis indicates that the primary feature dominating ferroelectricity is the tolerance factor that helps determine the paraelectric of chalcogenides perovskites, which will provide useful guidance for engineering them for suitable ferroelectricity.

The absence of thin-film samples of perovskite chalcogenide limits our basic knowledge of the optoelectronic properties and impedes their application to photovoltaic systems. The assembly of devices for solar cells depends basically on the properties of the thin films. Therefore, it is urgently vital to fabricate high-quality thin films and investigate their optoelectronic properties. Through the sputtering process, the new LaYS_3_ thin-film perovskite was developed. Therefore, the modification and optimization of the sputtering technique must be explored, and promising results must be applied to the manufacture of high-quality thin films of potential chalcogenide perovskites.

Meanwhile, considerable efforts have been made to fabricate the latest 3D lead-free chalcohalide perovskites of both MASbSI_2_ and MABiSI_2_ alongside their application in photovoltaic technologies—for instance, in solar cells; the recent focus has not been shifted to the fabrication of their 3D equivalents through compositional substitution and low dimensional chalcohalide perovskites via dimensionality tailoring. Accordingly, to develop new synthesis and characterization of novel lead-free chalcohalide materials with reduced dimensionality and compositional replacement for distinctive optoelectronic properties, compositional substitution, and dimensionality tailoring techniques must be incorporated into the formation of other lead-free chalcohalide perovskites. Lead-free chalcogenide and chalcohalide perovskites’ predicted benefits are nontoxic elemental composition and high thermal and moisture stability.

## Figures and Tables

**Table 1:** Summary of doped and undoped BaZrS_3_ Chalcogenide Perovskites.

Compound	Bandgaps (eV)	PL Peak (eV)	Synthetic Method	Reported by	Ref.
BaZr_1−x_TixS_3_	1.47		Heating BaS and ZrS_2_ in quartz tubes + 10% Ti-doped	Meng et al.	[24]
BaZrS_3_	1.82	1.81	BaZrO_3_ Thin film deposition by Pulsed laser deposition system and sulfurized with CS_2_	Wei et al.	[41]
Ba(Zr_1−x_Ti_x_)S_3_	1.51		Ball milling in steel jar a mixture of BaCO_3_, ZrO_2_, and TiO_2_ powders in stream of CS_2_	Wei et al.	[42]
BaZrS_3_	1.9	1.95	By chemical vapour deposition of BaZrO_3_ Thin film on quartz in stream of N_2_ and CS_2_	Pandey et al.	[43]
BaZrS_3_	1.8	1.84	By co-sputtering of BaS and Zr at ambient temperature to induce crystallization in streams of H_2_S	Comparotto et al.	[44]
BaZrS_3_	1.73	1.7	Heating BaZrO_3_ in quartz tubes under flowing Ar. And CS_2_	Perera et al.	[45]

**Table 2:** Summarized data for Chalcogenide of S and Se-based Photoabsorbers.

Compound	Bandgaps (eV)	PL Peak (eV)	Ref.
Ba_3_Zr_2_S_7_	1.28	1.28	[27]
CaSnS_3_	1.72		[46]
CaZrS_3_	1.90		[48]
SrZrSe_3_	1.02		[49]
LaYS_3_	2.0		[50]

**Table 3:** Summary of Transition Metal Perovskite Chalcogenides.

Compound	Bandgaps (eV)	PL Peak (eV)	Synthetic Method	Reported by	Ref.
α-SrZrS_3_	1.52	1.53	Iodine introduced as catalyst to solid reaction between SrS and Zr.	Niu et al.	[51]
β-SrZrS_3_	2.05	2.13			
BaZrS_3_	1.83	1.81	Iodine introduced as catalyst to solid reaction between BaS and Zr.	Niu et al.	[51]

**Table 4:** Summarised data for Bandgaps of undoped and doped Zr and Hf-based Distorted Chalcogenide Perovskites.

Compound	Measured Bandgaps (eV)	Calculated Bandgaps (eV)	Ref.
BaZrS_3_	1.94	1.77	[53]
SrZrS_3_	2.14	1.98	[53]
BaHfS_3_	2.17	2.01	[53]
SrHfS_3_	2.14	2.27	[53]
Ba(Zr_1−x_Ti_x_)S_3_	1.63		[53]
BaZr(S_1−x_Se_x_)_3_	1.76		[53]

**Table 5:** Summary of Hf-based Perovskite-Type Sulphide Photoabsorbers.

Compound	Bandgaps (eV)	PL Peak (eV)	Colour Emission	Ref.
SrHfS_3_	2.32	2.37	green	[55]
BaHfS_3_	2.06		orange	[55]

**Table 6:** Summary of device performance of Perovskite-based Chalcohalide photoabsorbers.

Compound	E_g_ (eV)	Voc (V)	J_sc_ (mA/cm^−2^)	FF	PCE (%)	Ref
MASbSI_2_	2.03	0.65	8.12	0.59	3.02	[66]
MABiSI_2_	1.52	0.22	1.96	0.30	0.13	[67]

## Data Availability

All the data is available within the manuscript.
